# Implant surface modifications and their impact on osseointegration and peri‐implant diseases through epigenetic changes: A scoping review

**DOI:** 10.1111/jre.13273

**Published:** 2024-05-15

**Authors:** Marcel F. Kunrath, Carlos Garaicoa‐Pazmino, Paula Milena Giraldo‐Osorno, Aya Haj Mustafa, Christer Dahlin, Lena Larsson, Farah Asa'ad

**Affiliations:** ^1^ Department of Biomaterials, Institute of Clinical Sciences, Sahlgrenska Academy University of Gothenburg Göteborg Sweden; ^2^ Department of Dentistry, School of Health and Life Sciences Pontifical Catholic University of Rio Grande do Sul (PUCRS) Porto Alegre Brazil; ^3^ Department of Periodontics University of Iowa College of Dentistry Iowa City Iowa USA; ^4^ Research Center, School of Dentistry Espiritu Santo University Samborondón Ecuador; ^5^ Institute of Odontology, Sahlgrenska Academy University of Gothenburg Göteborg Sweden; ^6^ Department of Oral Biochemistry, Institute of Odontology, Sahlgrenska Academy University of Gothenburg Göteborg Sweden

**Keywords:** bioactive surfaces, biomaterials, biomedical implants, bone‐implant interface, epigenomics, surface properties

## Abstract

Dental implant surfaces and their unique properties can interact with the surrounding oral tissues through epigenetic cues. The present scoping review provides current perspectives on surface modifications of dental implants, their impact on the osseointegration process, and the interaction between implant surface properties and epigenetics, also in peri‐implant diseases. Findings of this review demonstrate the impact of innovative surface treatments on the epigenetic mechanisms of cells, showing promising results in the early stages of osseointegration. Dental implant surfaces with properties of hydrophilicity, nanotexturization, multifunctional coatings, and incorporated drug‐release systems have demonstrated favorable outcomes for early bone adhesion, increased antibacterial features, and improved osseointegration. The interaction between modified surface morphologies, different chemical surface energies, and/or release of molecules within the oral tissues has been shown to influence epigenetic mechanisms of the surrounding tissues caused by a physical–chemical interaction. Epigenetic changes around dental implants in the state of health and disease are different. In conclusion, emerging approaches in surface modifications for dental implants functionalized with epigenetics have great potential with a significant impact on modulating bone healing during osseointegration.

## Graphical abstract

Implant surface modifications can trigger epigenetic mechanisms. In turn, epigenetic mechanisms can be utilized to functionalize implant surfaces to achieve better osseointegration.

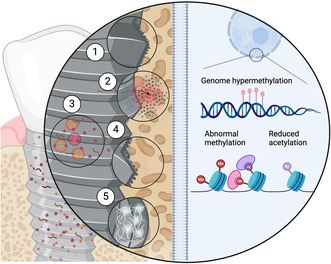

## 1 INTRODUCTION

Within the last three decades, surface modification of dental implants became a crucial factor for the enhancement of the osseointegration process and the reduction of early implant failures.^1^, ^2^ In recent years, several methods for surface modifications (e.g., texturization, hydrophilization, coatings, and functionalization with molecules/nanoparticles) have been proposed to accelerate osseointegration and simultaneously, provide an additional feature of resistance against bacterial contamination.^3^, ^4^, ^5^, ^6^ Most of these implant surface alterations are capable of modulating early extracellular and intracellular responses when the implant surface interacts directly with cells at the healing site.^7^, ^8^, ^9^

Recent studies have demonstrated that topographical, mechanical, and pharmacological stimuli of different biomaterials may influence gene expression.^10^, ^11^, ^12^ Epigenetics refers to alterations caused in the gene expression that are not encoded in the DNA sequence and thus, suggesting these changes correspond to the remodeling of the chromatin and the subsequent activation or inactivation of the gene expression.^13^ However, the effects of current surface modifications on the early and late epigenetics changes that may be associated with the osseointegration process remain unclear and not fully understood.

The potential role of implant surface topography and its modifications in promoting beneficial epigenetic changes is of paramount importance since it might influence the early and late stages of osseointegration^14^ and can lead to a better understanding of the prevention, pathogenesis and treatment success of peri‐implant diseases.^15^, ^16^ A preliminary study found that modified implant surface topographies can influence the DNA damage/repair pathway associated with epigenetic factors compared to non‐treated surfaces.^17^ Furthermore, it has been shown that human adipose‐derived stem cells (hACSs) cultivated on a nanotextured surface resulted in an increase of histone H3 at lysine 4 (H3K4) trimethylation levels and inhibited demethylase retinoblastoma binding protein‐2 (RBP2) expression.^10^ Such findings indicate the impact of surface modifications on epigenetic changes and its ability to influence stem cell fate.

The purpose of this scoping review is to summarize current commercially available and potential surface modifications of dental implants and the epigenetic changes induced upon peri‐implant cells and osseointegration. Lastly, this review highlights advances with possible future clinical implications in modern implant therapy.

## 2 SEARCH STRATEGY

Electronic and manual searches in PubMed, Medline, and EMBASE databases were performed until February 2024 to find reviews, in vitro, in vivo, experimental and/or clinical studies assessing the impact of surface modifications on peri‐implant cells and osseointegration of dental implants through epigenetic changes. The electronic search was executed using the following keywords and MeSH terms: “implants” or “dental implants” or “surface modifications” or “surface treatments” or “modified implants” and “epigenetic” or “DNA methylation” or “miRNA” or “histone modification” or “microRNA”. The inclusion criteria for the selected articles in this review considered: (1) English written studies, (2) literature/scoping reviews, (3) systematic reviews/meta‐analyses, (4) clinical trials, (5) animal, and (6) in vitro/in vivo studies. Two reviewers (M.F.K. and F.A.) evaluated individually the titles and abstracts of possible relevant studies to be addressed in this review. Then, the key studies were selected and investigated to summarize the data. Afterwards, the overall findings were described in a narrative style exploring the current “state of the art” on this topic.

## 3 SURFACE MODIFICATIONS OF INNOVATIVE AND MODERN COMMERCIALLY AVAILABLE DENTAL IMPLANTS

In the last decade, technological advances in biomaterials have led to an exponential increase in the development of novel surface treatments for dental implants targeting improved bone–implant interface healing in patients.^18^, ^19^ Surface modifications aiming for physical, chemical, and pharmacological changes have been proposed to improve the early and late responses to the osseointegration process of dental implants.^1^, ^3^, ^5^, ^7^, ^20^ Currently, properties such as roughness, wettability, surface morphology, crystallinity, and surface bioactivity have been shown to affect the cellular response at several levels and especially, by promoting distinct tissue behaviors and a faster healing.^3^, ^5^, ^7^, ^20^, ^21^, ^22^

**FIGURE 1:**
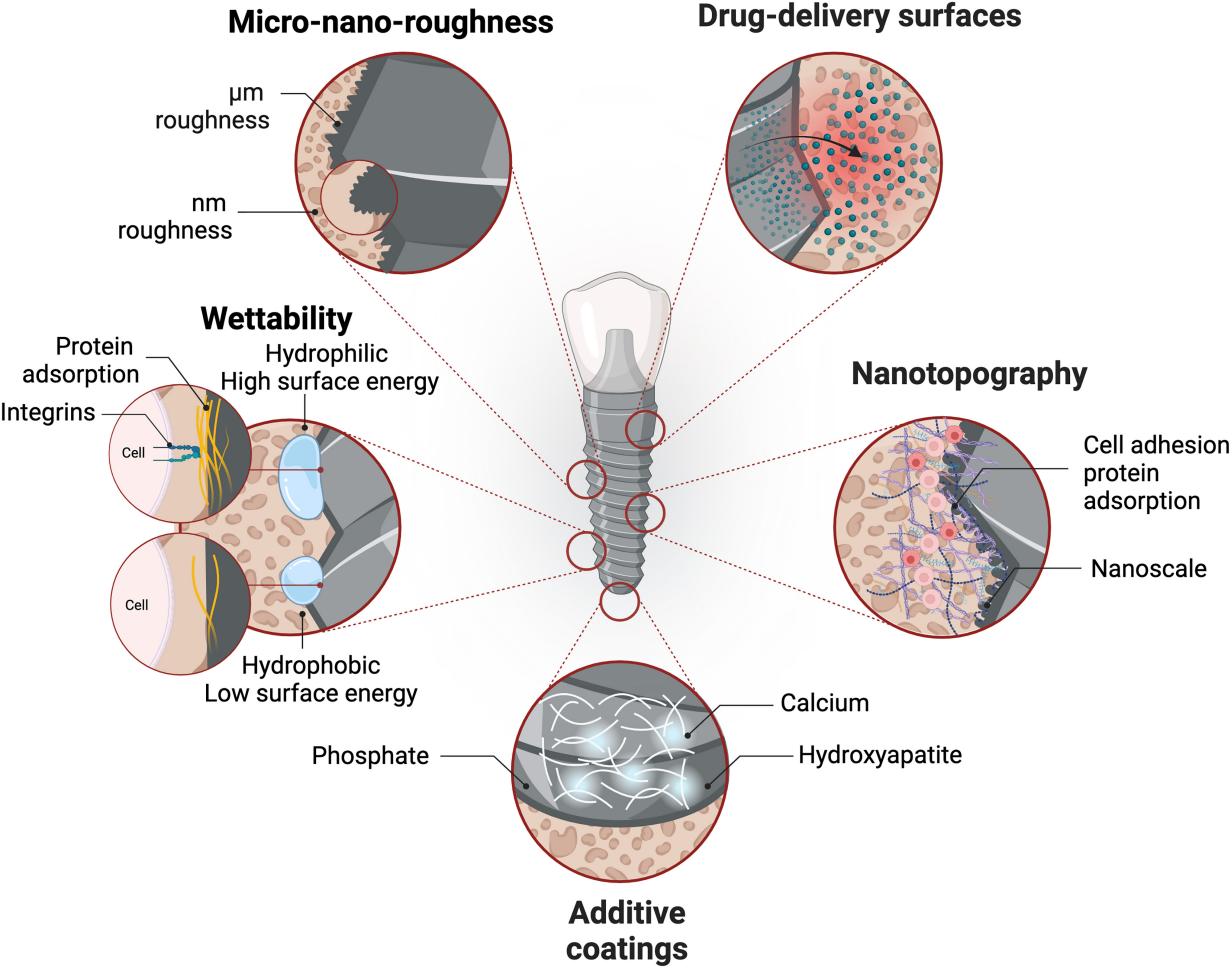
Illustration showing examples of several innovative modifications for dental implant surfaces.

Modern dental implants use a combination of several modified properties within the same implant surface resulting in better bone‐to‐implant contact (BIC) and/or accelerating the osseointegration process suggesting the feasibility of supporting clinical prosthetic loading at earlier stages of healing.^23^ Most of these altered surface properties affect biological processes occurring at very early stages of osseointegration in the implant–bone interface at a molecular level, and usually, immediately after surface exposure to cells and tissues. Additionally, it is difficult to evaluate surface modification effects on long‐term osseointegration due to the requisite to perform long‐term preclinical studies or aggressive clinical sampling of osseointegrated implants. Hereby, a brief explanation of the outcomes related to these surface modifications is available below in addition to the specific impact of each innovation upon early and late stages of osseointegration (Figure 1).

### 3.1 Micro‐ and nano‐roughness

Historically, surface roughness was the first property altered in dental implant surfaces aiming for better osseointegration by modifying polished into rougher surfaces.^24^ The outcomes of this alteration were extremely significant as it promoted faster healing periods and early implant loading. Moreover, these changes in implant surface roughness not only resulted in improved osseointegration but were particularly beneficial for sites with poor bone quality.^2^, ^24^, ^25^

Supporting these statements, one of the largest retrospective studies with 27 years of implant survival follow‐ups, demonstrated the positive impact of rougher surfaces compared with smooth surfaces on the rates of implant survival after long‐term osseointegration.^2^ Furthermore, rougher implant surfaces have been demonstrated to reduce the number of failures after the delivery of implant‐retained prosthesis.^26^

Advances in surface treatment methodologies, coupled with emerging research findings, confirmed the influence of micro‐ and nanotopography on the proliferation, adhesion, viability, and mineralization of cells involved in the osseointegration process.^4^, ^6^, ^7^, ^8^, ^18^, ^27^ The surface area is enlarged by micro‐ and nano‐valleys or peaks increasing the actual surface interaction with serum proteins and mesenchymal/bone‐related cells, and consequently, inducing augmented bone deposition surrounding the entire implant surface.^28^ It has been shown that micro‐ and nano‐roughness significantly improved the levels of BIC and removal torque of dental implants.^29^, ^30^ Several current methodologies have been proposed to modify dental implant roughness, for instance, the majority reported in experimental and clinical studies include sandblasting, acid‐etching, combinations of sandblasting and acid‐etching, anodization, and laser texturing.^18^

On the other hand, it is important to report that implant surfaces with elevated roughness parameters have been shown to facilitate bacterial colonization and biofilm formation both for titanium or zirconium surfaces^31^ and thus, leading to an increased susceptibility for peri‐implant diseases.^32^

In summary, micro‐ and/or nano‐roughness modifications have been revealed to improve biocompatibility and promote faster and improved osseointegration, albeit with a relative risk of an increased bacteria adhesion on implant surfaces presenting high rougher characteristics.

### 3.2 Wettability

In recent years, innovations in the techniques for the hydrophilization of dental implants have been of particular interest to implant companies. Novel processes applying ultraviolet lights exposure,^33^ plasma treatments,^20^ heating methods,^34^ and chemical modifications,^35^ led to the creation of highly hydrophilic surfaces. These methodologies generate chemical modifications at the implant surface promoting the creation of terminal radical groups (−OH) freely to bind to any external molecules and allied with the induction of a higher surface energy.^36^, ^37^ Conversely, surface energy may be altered easily by changes in topographic morphology, roughness parameters, or by the addition of external coatings with another biomaterial deposition, stimulating different chemical exchange with fluids underlying the surface, or generating low‐energy surfaces.^36^

Differently from the alterations caused by changes in roughness characteristics and surface area, variability in surface energy may directly affect the interaction with bone‐related cells on the molecular level, inducing the expression of important genes related to new bone formation, while a large surface area increases the physical space for cell adhesion and proliferation. The combination of these physiochemical modifications on the same implant surface may be promising for positive intracellular upregulation of bone factors (e.g. alkaline phosphatase [ALP] and osteocalcin) promoting a better early osseointegration process.^37^

The impact of hydrophilic surfaces is revealed to be of great significance at the early stages of osseointegration as these might induce the spread of mesenchymal and bone‐related cells over the implant surface and the adsorption of proteins to the surface. As a result, a quick immunological cascade is triggered, thus, accelerating the healing process.^33^, ^34^, ^38^

Additionally, hydrophilic surfaces have shown to be quite sensitive to several environmental factors such as the interaction with oxygen^35^ and contact with saliva.^27^ These findings suggest the necessity of more rigid care in the handling and translation of hydrophilic surfaces into real clinical situations. Nevertheless, this property has proven to influence early stages of osseointegration and show enhanced outcomes for dental implants that aim for faster healing among in vitro, preclinical, and clinical studies.^20^, ^33^, ^38^

Ultimately, it has been suggested that the time for final prosthesis delivery after implant placement is reduced using dental implants with hydrophilic surfaces due to the quick bone‐implant interface maturation.^38^ Nonetheless, the complete process and the advantages of hydrophilic surfaces in the early and late stages of osseointegration are not fully elucidated by robust molecular, histological, and mechanical data and deserve further studies.

### 3.3 Nanotopography

The concept of nanotopography is related to a whole surface presenting nano‐scaled morphology. Research has shown the potential of nanofeatures in several parameters related to the osseointegration process in the early stages of healing including superior cellular behavior,^7^, ^20^ antibacterial characteristics,^39^ and orientation of soft tissue cells.^40^

For instance, bone‐related cells exhibited promising morphology and higher adhesion to nanostructured surfaces compared with macro and micro‐structuration demonstrating advanced filopodia within the implant surface, allied with reduced taxes of *Staphylococcus epidermidis* adhesion in the early stages of investigation.^22^

Additionally, these surfaces can increase cellular interaction and expression of bone‐related genes associated with osseointegration when nanotextured surfaces are applied in vivo^8^, ^41^ and when combined with noble metal coating, nanostructured surfaces reduced the number of bacterial colonies compared with non‐coated surfaces demonstrating osseointegration characteristics similar to non‐treated surfaces.^42^ Similarly, Bright et al. developed a nanostructured surface to test in a long term (21 days) in vitro model evaluating the antibacterial properties of this surface revealing a reduced bacterial viability, lower biovolume accumulation, and eventual membrane damage in bacterial cells exposed to nanosurfaces compared to polished (control) surfaces.^43^

Ultimately, Gulati et al. highlighted the promising feature of promoting alignment and guidance of human gingival fibroblasts using nanotubular surfaces.^40^ The authors concluded that the nanotubes orientation might induce fibroblasts to spread and proliferate in one specific direction, and thus, increasing cell adhesion properties and suggesting optimistic soft tissue sealing in trans‐mucosal implant components.

Osseointegration outcomes comparing the application of nanostructured and microstructured implant surfaces in a rabbit model after 4 weeks of healing revealed similar bone anchorage for both surfaces, however, slightly better results for the nanosurface considering BIC and bone area parameters.^44^ These findings suggest that the effects of surface nanostructure may be expressively higher at early stages of healing and similar to other surfaces in late osseointegration.

Despite the fact that nanostructured surfaces may support better osseointegration conditions compared with the currently available dental implant surfaces, the protocols for the development of flawless nanomorphologic surfaces are not elucidated and further translational studies are needed before their use in patient care.

### 3.4 Coated surfaces

Unlike subtractive techniques, additive processes have the intention to create surfaces mimicking bone characteristics. Coated surfaces use chemical combinations like calcium, phosphate, and hydroxyapatite due to their biocompatibility and excellent outcomes throughout and beyond the healing stages of osseointegration.^45^, ^46^, ^47^

The application of a new layer attached to the implant surface can change properties such as biocompatibility, corrosion resistance, and mechanical features.^48^, ^49^ Techniques such as plasma spray have shown to create texturized coatings demonstrating higher cell adhesion and proliferation, thus, suggesting improvement in bone quality in the osseointegration process.^48^

Additively coated implant surfaces should demonstrate excellent characterization of the coating adhesion and resistance to high torque insertions. Nevertheless, the coating adhesion to the substrate must be rigid so as not to rupture during the implant insertion. A coating rupture can release metal‐like fragments or particles to the peri‐implant tissues and elicit detrimental effects.^50^, ^51^ Therefore, the long‐term results of osseointegration of coated implant surfaces should demonstrate better outcomes compared to non‐coated surfaces.

Recently, not only inorganic coatings have been proposed for dental implants but a large spectrum of organic coatings have been suggested, by applying synthetic or natural materials (e.g., poly lactic‐*co*‐glycolic acid (PLGA), polyethylene glycol (PEG), polysaccharides (chitosan), collagen and extracellular matrix proteins, autologous platelet concentrates).^52^ These biomaterials have shown degradable properties and biocompatible features which may induce improved healing surrounding the peri‐implant tissues. Organic coatings may carry relevant molecules to the process of new bone formation aiming at accelerated healing process on the interface of dental implants, for example, natural coatings have a quick biodegradable process stimulating fast release of their elementary composition into the surrounding tissues. On the contrary, PLGA or PEG may support a sustainable release of incorporated molecules with biocompatibility.^52^, ^53^ Nevertheless, innovative coatings need optimization and several advances in preclinical studies to progress to the clinical market.

### 3.5 Drug‐release surfaces

A new promising technology has been explored by associating diverse processes for surface modifications with molecules and/or drug delivery.^54^ The term “smart surfaces” has been used for surfaces that may perform several biological functions due to their multifunctional properties and localized application.

Micro‐nanotopographies, coated surfaces, or combinations of these surface treatments have been associated with pharmacological molecules (e.g., analgesics, anti‐inflammatories, antibiotics, osteo‐promoters, natural molecules, among others) for the local delivery upon the healing interface of dental implants aiming for antibacterial function, accelerated bone formation, and reduction in the inflammatory process.^5^, ^53^, ^54^, ^55^, ^56^ This drug‐delivery property has been shown to accelerate the proliferation and adhesion of bone‐related cells, in addition to modulating the molecular expression of bone‐related genes. Gene expression of osteogenic cells and viability was increased when associated with osteoinductive molecules (e.g., bone morphogenetic proteins [BMP]) and have been demonstrated to improve antibacterial properties when paired with antibiotics and/or peptides.^55^, ^56^, ^57^

A systematic review reported that local drug delivery of calcium phosphate, bisphosphonates, and BMPs on the interface of dental implants inserted within in vivo models were demonstrated to improve the osseointegration process without eliciting side effects (e.g., cytotoxicity).^58^ Additionally, antibiotic‐loaded surfaces (amoxicillin) applied in experimental models were protective against the bacterial contamination in the in vitro conditions with high efficacy compared with standard surfaces.^59^ The combination of two or more molecules within the same surface is possible as these can stimulate different targets of the osseointegration process.^57^, ^58^ The locally controlled release of molecules by modified surfaces may prevent early implant loss and also, improve the environment for osseointegration, especially among patients with compromised health conditions affecting wound healing and bone regeneration.^60^

These pharmacological devices have a long pathway to being approved as commercial products. Multiple requirements are necessary to ensure safety and product approval starting from the material basic production to the traceability after the product selling.^61^

However, dental implants equipped with drug‐delivery systems, innovative coatings, and complete nanotexturization are not commercially available in the dental market. Results from basic and preclinical research are promising and the inherent emergence of these surfaces in clinical practice can become revolutionary in dentistry and implant therapy.

## 4 THE IMPACT OF DENTAL IMPLANT SURFACE MODIFICATIONS UPON PERI‐IMPLANT CELLS AND OSSEOINTEGRATION THROUGH EPIGENETIC CHANGES

Epigenetic mechanisms encompass a range of processes that regulate gene expression beyond the DNA sequence itself.^62^, ^63^ These mechanisms include DNA methylation, histone modifications, and microRNAs (miRNAs). DNA methylation involves the addition of methyl groups to the 5′ position at the base cytosine (5mC), resulting in gene silencing.^62^ Histone modifications refer to post‐translational changes in histone proteins, such as acetylation, methylation, and phosphorylation, which impact chromatin structure and gene accessibility.^64^ Ultimately, microRNAs are small non‐coding RNAs that regulate gene expression post‐transcriptionally by binding to target mRNAs, resulting in the suppression of gene expression, either by degradation of a target messenger RNA (mRNA) or by preventing translation.^65^

The structure and energy of a surface have been shown to influence chromatin formation. Cells grown on a low‐energy surface (e.g., a soft material) display a transcriptionally inactive chromatin, while cells expanded on a high‐energy surface (e.g., a stiff material) have a transcriptionally active chromatin.^13^, ^66^ The underlying mechanisms involve the connection of the nucleus and its chromatin to the extracellular matrix through a complex network of extracellular and intracellular fibers. These intricate networks are highly responsive to the surrounding environment and surfaces with which cells interact.^67^ The TopoChip model enables the screening of many surface micro topographies. This model has shown that surface topography does exert influence over nuclear shape and content, along with histone modifications. Interestingly, these observed changes were found to be reversible when cells initially grown on surface topography and subsequently, re‐cultured on flat surfaces.^68^, ^69^

### 4.1 Epigenetic changes upon bone cells and osseointegration

**FIGURE 2:**
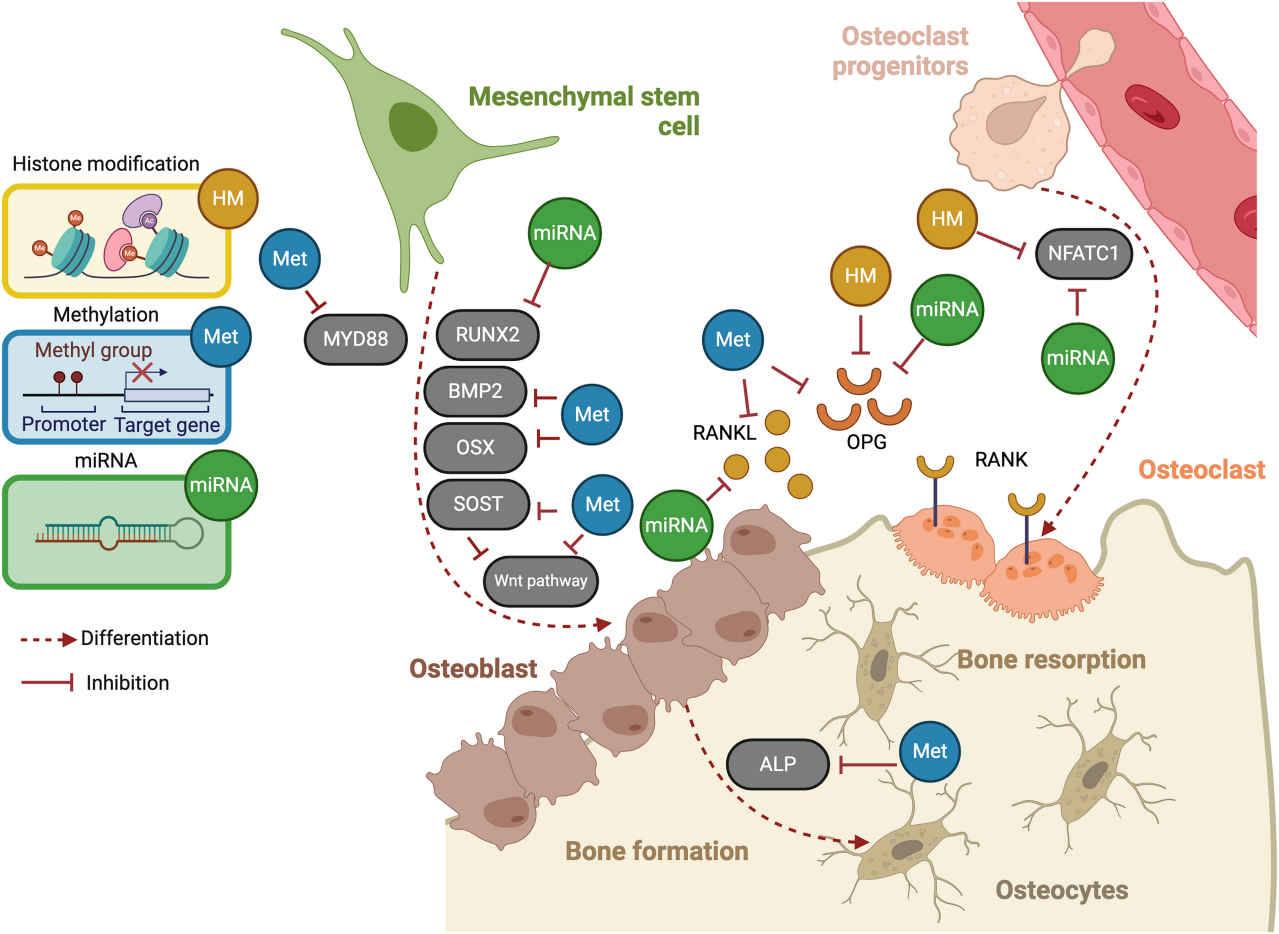
Epigenetic regulations in bone metabolism. DNA methylation maintains the balance between OPG and RANKL; hypermethylation of the BMP2 promoter in osteoblasts downregulates bone formation markers. Similarly, hypermethylation of sclerostin (SOST) inhibits SOST gene translation, favoring osteoclast differentiation. Histone modifications inhibit osteoclast differentiation through the nuclear factor of activated T‐cell cytoplasmic 1 (NFATC1) gene. MiRNAs modulate the expression of RUNX2 and NFATC1, influencing both osteoblast and osteoclast differentiation.

A myriad of studies^17^, ^70^, ^71^, ^72^, ^73^, ^74^, ^75^, ^76^, ^77^, ^78^, ^79^, ^80^, ^81^ explored the expression of microRNAs, histone acetylation/methylation, and DNA methylation in preclinical and experimental studies and their impact on stem cells, bone tissue and osseointegration. The different epigenetic mechanisms have been shown to affect osteogenesis and osteoclastogenesis, which is relevant to osseointegration around dental implants (reviewed in Asa'ad, Monje & Larsson 2019^82^) (Figure 2).

In addition to regulating chromatin configuration, cell contact with titanium has been shown to activate the DNA damage pathway.^66^, ^83^ An in vitro study using titanium discs with various surface properties showed that these not only influenced DNA damage but also impacted DNA repair pathway and epigenetic factors.^17^

Furthermore, titanium discs with a rougher surface induced cytoplasmic staining of DNA methyltransferase‐1 (DNMT1), while presenting a lower percentage of acetylated histone‐3(AcH3)‐positive osteoblastic cells compared with glass and smooth titanium surface.^17^ Cho and co‐workers showed that pre‐osteoblasts grown on titanium with sandblasted, large‐grit acid‐etched (SLA) surface had a lower methylation level of the ALP gene compared with cells grown on machined titanium surfaces, increasing ALP expression.^78^ Similarly, Lyu and co‐workers found an increase of methylation in genes, such as ALP, involved in osteogenic mechanisms in human bone marrow mesenchymal stem cells (hBMSCs) grown on SLA titanium surface compared to smooth titanium.^76^ This was also shown for genes associated with the Wnt and cadherin signaling pathways. Interestingly, genes involved in these pathways were found to differ in their methylation pattern. Out of a total of 2846 CpG sites differently methylated in SLA compared with smooth titanium, 1651 CpG sites were found to be hypermethylated and 1195 were hypomethylated in the SLA group.^76^

Topographical cues of titanium surfaces have the potential for the induction of osteogenic differentiation of BMSCs through regulation of the level of H3K4me3 and H3K27me3 at the Runt‐related transcription factor 2 (RUNX2) promoter.^75^ H3K4me is associated with gene expression while H3K27me3 is responsible for silencing of gene expression.^84^ Growing BMSCs on titanium discs treated with direct metal laser sintering (DMLS) technique and comparing the osteogenic differentiation to cells grown on SLA and smooth titanium has shown that the DMLS surface was more favorable for osteogenic differentiation.^75^ It was suggested that this increase is associated with H3K27 demethylation and an increase in H3K4me3 at the RUNX2 promoter. The DMLS technique rendered a surface with a hierarchical micro/nanoscale topography, closely resembling that of bone, not only to enhance cell alignment but also to modify nucleus shape. Furthermore, it has been found to influence the level of acetylated histone‐4 (AcH4), AcH3, and H3K9me3 expression.^85^

Osteoblasts grown on titanium discs with a nanotopography surface showed a similar expression of H3K9me2, H3K27me3, and enhanced of zeste homolog‐2 (EZH2) as to cells grown on an untreated titanium surface when co‐cultured with osteoclasts.^79^ In contrast, it was also reported that the accumulation of H3K27me3 in the gene promoters of RUNX2 and ALP resulted in the suppression of those genes and prevented cell growth on titanium nanosurfaces in the presence of osteoclasts. Thus, it was suggested that the epigenetic mechanisms triggered by a surface with a nanotopography may protect osteoblast from the deleterious effects of osteoclasts and improve osseointegration of implants.^79^

Only a few studies have investigated the influence of biomaterials on miRNA expression.^70^, ^71^, ^81^ Osteoblast‐like cells (MG63) grown on titanium discs and culture plates showed a significant difference in miRNA expression.^70^, ^71^ When these cells were grown on grade 3 titanium and zirconium dioxide ceramics (ZDCs) discs, 6 miRNAs were upregulated (miR‐214, miR‐337, miR‐423, miR‐339, miR‐377, and miR‐193b) and 4 were downregulated (miR‐143, miR‐17‐5p, miR‐24, and miR‐22) in cells grown on ZDCs compared to cells grown on titanium.^70^ Conversely, BMP4 and BMP7 were upregulated in cells grown on titanium compared with those grown on ZDCs.

**FIGURE 3:**
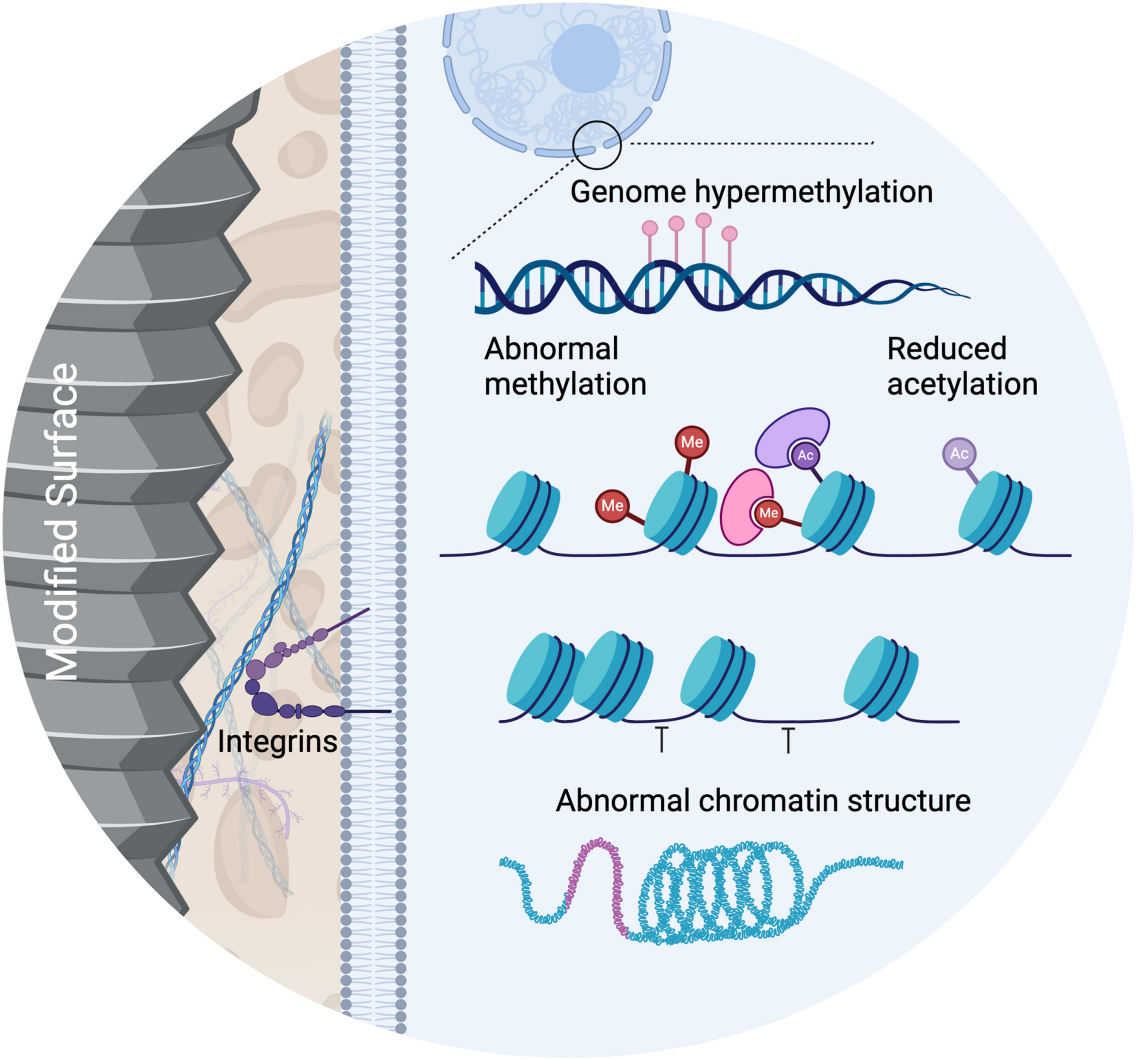
Epigenetic mechanisms triggered by implant surface modifications such as histone acetylation/methylation and DNA methylation. Ac, acetylation; Me, methylation.

It should be noted that the results and variations between studies on the effect of surface structure and surface energy need to be carefully interpreted. Multiple factors and their microenvironment can influence the results of an in vitro study including, but not limited to, cell types, cell culture substrate, cell culture medium and cell growth in 2D cell cultures on flat surfaces respond differently to those that grow on 3D structures.^86^ A summary of the potential epigenetic mechanisms in bone triggered by implant surface modifications is depicted in Figure 3. Summary of the studies on the effect of implants on epigenetics in bone cells is displayed in Table 1.

**TABLE 1:** Characteristics of studies evaluating implant surface modifications and their epigenetic changes upon bone cells and osseointegration.

Author	Type of study	Experiment	Type of implant surface	Epigenetic mechanism	Method	Findings
Palmieri et al.^70^	In vitro	MG‐63 cells were cultured on titanium and zirconia disks	Grade 3 Titanium (Machined) and Zirconia dioxide ceramic	MicroRNA expression	MicroRNA microarray	Compared with Titanium exposure of MG‐63 cells to Zirconia‐induced upregulation of 6 miRNAs (miR‐214, miR‐337, miR‐423, miR‐339, miR‐377, and miR‐193b) and downregulation of 4 miRNAs (miR‐143, miR‐17‐5p, miR‐24, and miR‐22). Potentially, a higher osseointegration capacity was detected for Titanium surfaces compared with titanium.
Palmieri et al.^71^	In vitro	MG‐63 cells were cultured on zirconium oxide disks	Zirconium oxide	MicroRNA expression	MicroRNA microarray	Exposure of MG‐63 cells to zirconia‐induced upregulation of 18 miRNAs (miR‐337, miR‐423, miR‐497, miR‐214, miR‐377, miR‐296, miR‐99b, miR‐193b, miR‐25, miR‐324, miR‐518a, miR‐320, miR‐23b, miR‐93, miR‐23a, miR‐422b, miR‐330, and miR‐197) and downregulation of 3 miRNAs (miR‐302c, miR‐369‐5p, and miR‐10b)
Palmieri et al.^81^	In vitro	MG‐63 cells were cultured on titanium disks	Titanium with anatase coating	MicroRNA expression	MicroRNA microarray	Exposure of MG‐63 cells to anatase coating induced upregulation of 9 miRNAs (miR‐19b, miR‐198, miR‐34c, miR‐210, miR‐132, miR‐29a, miR‐27a, miR‐18a, and miR‐205) and downregulation of 10 miRNAs (miR‐148a, miR‐93, miR‐324, miR‐494, miR‐103, miR‐1, miR‐22, miR‐23b, let‐7 g, and miR‐422b).
Chakravorty et al.^72^	In vitro	Primary human osteoprogenitor cells cultured on titanium disks	Grade 2 titanium with Smooth, SLA and modified SLA	MicroRNA expression	Quantitative real‐time PCR	Compared with smooth surfaces, 39 miRNAs were downregulated and 11 were upregulated on modified SLA surfaces whereas 38 were downregulated and 10 were upregulated in SLA surfaces. Several potential osteogenic genes were identified for miR‐215 and miR‐125b. Expression of miRNAs influences genetic mechanisms leading to osteogenic differentiation of modified titanium implant surfaces.
Wimmers‐Ferreira et al.^74^	In vitro	Human alveolar bone cells cultured on titanium surfaces	Titanium with nanotexture, nano/submicrotexture and rough microtexture	MicroRNA expression	MicroRNA microarray/hybridization, quantitative real‐time PCR	Oxidative nanopatterning of titanium surfaces induces changes in metabolism of osteoblastic cells. Differential expression of 716 mRNAs and 32 microRNAs with functions associated with osteogenesis was observed.
Iaculli et al.^73^	In vitro	Dental pulp stem cells cultured on titanium disks	Sandblasted and acid‐etched	MicroRNA expression	Real‐time PCR	MiR‐133 and miR‐135 played a pivotal role in the osteogenic differentiation of MSC. Modified surfaces (sandblasted and acid‐etched surfaces enhanced the development of osteoblasts cells and thus, leading to a faster osseointegration process.
Zheng et al.^75^	In vitro	In vitro: BMSC exposed to titanium disks. In vivo: Ectopic BMSC‐coated disks were implanted in Sprague–Dawley rats	Titanium treated with direct metal laser sintering, SLA and smooth surfaces	Histone acetylation	Real‐time PCR, Immunofluorescence	DMLS surface is more favorable for osteogenic differentiation of bone marrow MSCs than SLA and smooth Titanium surfaces. Rapid H3K27 demethylation and increase in H3K4me3 levels at gene promoters upon osteogenic differentiation were observed DMLS titanium surfaces.
Lyu et al.^76^	In vitro	Human BMSC exposed to titanium slices	Titanium with SLA and machined surfaces	DNA methylation	Illumina Infinium Methylation	2846 CpG sites were differentially methylated in the SLA surface compared with machined surfaces. Among them, 1651 were hypermethylated and 1195 were hypomethylated. 37 genes displayed a negative association between mRNA expression and DNA methylation level. SLA and machined surfaces have different effects on genome‐wide DNA methylation and enrichment of osteogenic pathways among human BMSC.
Zhuang et al.^77^	In vitro	BMSCs exposed to titanium disks	Grade 2 nanostructured titanium	MicroRNA expression	Quantitative real‐time PCR, Transfection	Elevated miR‐23 inhibited osteogenic differentiation process of BMSC and decreased miR‐23 levels enhanced this process.
Cho et al.^78^	In vitro	Mouse pre‐osteoblast MC3T3‐E1 cells seeded to titanium disks	Titanium with SLA and machined surfaces	DNA methylation	Real‐time PCR	The degree of DNA methylation on the ALP gene was lower on SLA than machined surfaces. DNA methyltransferase inhibitor stimulated the ALP gene expression on machined surfaces similar to SLA surfaces. A superior osteogenic potential of SLA surfaces ca be attributed to a different epigenetic landscape, specifically, the DNA methylation of ALP genes.
Ichioka et al.^17^	In vitro	MG‐63 cells were cultured on titanium disks	Glass and Titanium (smooth and minimally rough) surfaces	DNA damage, histone acetylation and DNA methylation	Immunofluorescence	Minimally rough titanium surfaces induced cytoplasmic staining of DNMT1 up to 99% at 24 hours and showed the lowest percentage of AcH3‐positive cells compared with glass and smooth titanium surfaces.
Bighetti‐Trevisan et al.^79^	In vitro	Pre‐osteoblast MC3T3‐E1 cells seeded to titanium disks	Titanium with non‐conditioned and nanotopography	Histone methylation	Real‐time PCR, Chromatin immunoprecipitation, RNA‐seq, Immunofluorescence	Osteoclasts downregulated expression of osteoblast markers genes and upregulated genes related to histone modifications and chromatin organization in osteoblasts grown on both surfaces. Osteoclasts increased the expression of H3K9me2, H3K27me3 and EZH2 in osteoblasts grown on both surfaces. Osteoclasts increased the accumulation of H3K27me3 that represses the promotor regions of RUNX2 and ALP in osteoblasts on non‐conditioned titanium surfaces and reduced in titanium with a nanotopography surface. Nanotopography attenuates the osteoclast‐induced disruption of osteoblast differentiation by preventing the increase of H3K27me3 accumulation that represses the promoter region of key osteoblast marker genes.
Fernandes et al.^80^	In vitro	Human endothelial cells exposed to a titanium‐enriched medium	Titanium with dual acid‐etching and machined surfaces	Histone acetylation and DNA methylation	Quantitative real‐time PCR	Histone deacetylases and Sirt1 drives chromatin condensations whereas DNMTs and TET methylcytosine dioxygenases conducts the methylation profile of DNA strands. HDAC6 emerges as an important player in the epigenetic mechanism in endothelial cells. Titanium maintains the surrounding microenvironment dynamically active and affects the performance of endothelial cells by modulating epigenetics, specifically, HDAC6.

Abbreviations: AcH3, acetylated histone‐3; ALP, alkaline phosphatase; BMSC, bone marrow stem cells; DMLS, direct metal laser sintering; DNMT1, DNA methyltransferase‐1; EZH2, enhanced of zeste homolog‐2; HDAC6, histone deacetylase‐6; miRNA, microRNA; MSC, mesenchymal stem cells; PCR, polymerase chain reaction; RUNX2, runt‐related transcription factor‐2; Sirt1, sirtuin‐1; SLA, sandblasted, large‐grit, acid‐etched; TET, ten‐eleven translocation.

### 4.2 Epigenetic changes upon the peri‐implant mucosa and liquid samples

Understanding the epigenetic regulations at the peri‐implant soft tissues is crucial as tissue homeostasis at the epithelial‐connective tissue interphase preserves the integrity of osseointegration of dental implants. However, limited evidence is available attempting to establish a correlation of the effects of implant surface modifications upon peri‐implant tissue homeostasis, pathogenesis, and treatment of peri‐implant diseases. MiRNA expression was evaluated in soft tissue biopsies from ligature‐induced peri‐implantitis sites around SLA implants in dogs.^87^ The miRNA expression profile showed that let‐7 g, miR‐27a and miR‐145 might play specific roles in inflammation and osteoclastogenesis during peri‐implantitis. In a follow‐up experimental study,^88^ the authors demonstrated that treatment of peri‐implantitis defects with miR‐27a promoted re‐osseointegration in the regenerative treatment of peri‐implantitis. As such, miRNA‐based approach may be a valuable tool for improving guided bone regeneration (GBR) outcomes during peri‐implantitis treatment.

A prospective case series using a split‐mouth design among 10 patients receiving dental implants with a fully dual acid‐etched surface (DAE) and hybrid surface (DAE and machined at the most coronal part of the implant) showed differences in miRNAs expression and clinical parameters in soft tissues around dental implant for up to 5 years.^89^, ^90^ A total of 96 miRNAs were differently expressed between these two implants and displayed a greater difference in expression pattern between sites with and without bleeding on probing.

Regarding DNA methylation on soft tissues, Khouly et al. assessed in a cross‐sectional study the global DNA methylation in soft tissues and bone, harvested from failed dental implants.^91^ The results showed similar global DNA methylation levels between peri‐implant health and failed dental implants due to peri‐implantitis. Moreover, higher DNA methylation levels were found in soft tissues when compared to bone, which confirms the specificity of epigenetic changes to the microenvironment. Since the type of dental implant surface was not specified in the study, no connection between implant surface and peri‐implantitis pathogenesis was established.

The possible association between miR‐146a/miR‐499 gene polymorphisms and peri‐implantitis was investigated in serum samples in an Iranian population.^92^ Although the authors concluded that miR‐146a/miR‐499 gene polymorphisms might be genetic determinants for increased risk for peri‐implantitis, an association between implant surfaces and peri‐implantitis pathogenesis cannot be stated due to missing information on the type of implant surfaces.

Daubert et al (2019) evaluated global DNA methylation and quantified titanium particles in peri‐implant crevicular fluid (PICF) from peri‐implantitis‐affected and healthy implants.^93^ Their results revealed an increased global DNA methylation level in peri‐implantitis cases. Interestingly, higher global DNA methylation levels correlated with higher titanium quantities in PICF, irrespective of the peri‐implant status, which might suggest the influence of titanium particles on DNA methylation. It must be noted that the implant surfaces were not specified in this study as well, and thus, it is not possible to extrapolate the association between implant surfaces and global DNA methylation.

A summary of the studies on the effect of implants on epigenetics in soft tissues and liquid samples is displayed in Table 2.

**TABLE 2:** Characteristics of studies evaluating implant surface modifications and their epigenetic changes among liquid samples and peri‐implant tissues.

Author	Type of study	Description/condition of sites	Type of implant brand/surface	Epigenetic mechanism	Method	Findings
Kadkhodazadeh et al.^92^	Case control	Serum samples among patients with chronic periodontitis, peri‐implantitis, and periodontal health	N/A	MicroRNA gene polymorphism	Competitive allele‐specific PCR	MiR‐146 and miR‐499 gene polymorphisms may be genetic determinants for an increased risk for periodontitis and peri‐implantitis. No connection between surface and pathogenesis was established
Wu et al.^87^	Experimental	Soft tissue biopsies of peri‐implant healthy and ligature‐induced peri‐implantitis sites for 2 months among 6 Labrador dogs	Straumann, SLA	MicroRNA expression	Quantitative real‐time PCR	let‐7 g, miR‐27a and miR‐145 might play specific roles in inflammation and osteoclastogenesis during peri‐implantitis. No connection between surface and pathogenesis was established
Daubert et al.^93^	Case control	PICF from healthy and peri‐implantitis‐affected implants among 40 patients	N/A	DNA methylation	Global DNA methylation assay	Levels of methylated DNA cytosine (5mC) was significantly more pronounced in peri‐implantitis compared to peri‐implant health. DNA methylation may be affected by titanium dissolution products. No connection between surface and pathogenesis was established
Menini et al.^89^	Prospective case series	Soft tissue biopsies were collected after 3 months of implant placement (Osseotite, Biomet 3i) among 7 patients	Biomet 3i, Hybrid (Dual acid‐etch and machined)	MicroRNA expression	MicroRNA microarray	After 5 years, miR‐33, miR‐134, miR‐200 and miR‐378 were predictors for bone resorption and playing a regulatory role in cell proliferation, stem cell recruitment/differentiation, osteogenesis, and inflammation. Similarly, miR‐517, miR‐525, miR‐624, miR‐3128, miR‐3658, miR‐3692, miR‐3912, miR‐3920, miR‐4683, miR‐4690 served as predictors for peri‐implantitis regulating inflammation, cellular proliferation, and cartilage homeostasis. Proposed use of protective miRNAs as possible drugs/coating for implant surfaces.
Wu et al.^88^	In vitro, Experimental	In vitro: BMSCs. Experiment: Ligature experimental peri‐implantitis was used to treat bony defects with B‐TCP alone, B‐TCP + BMSCs, BMSCs transduced with Lenti‐miR‐NC or BMSCs transduced with Lenti‐miR‐27a among 5 Labrador dogs	Straumann, SLA	MicroRNA expression	In vitro: Quantitative real‐time PCR, Western blot, tube formation assay, dual‐luciferase reporter assay. Experimental: Micro‐CT, histology	In vitro: Overexpression of miR‐27a positively regulated osteogenesis‐angiogenesis coupling by ameliorating the TNF‐a inhibition of bone formation in vitro. Experimental: MiR‐27a targets Dickkopt2 and SFRP1 to promote re‐osseointegration in the regenerative treatment of peri‐implantitis. miRNA‐based approach may be a valuable tool for improving GBR outcomes during peri‐implantitis treatment.
Khouly et al.^91^	Cross‐sectional	Soft and hard tissue biopsies were collected from periodontally healthy and peri‐implantitis affected implants among 19 patients	N/A	DNA methylation	Global DNA methylation assay	Similar global DNA methylation levels were observed between peri‐implant health and peri‐implantitis. Higher DNA methylation levels were found in soft tissues when compared to hard tissues. No connection between surface and pathogenesis was established

Abbreviations: BMSC, bone marrow stem cells; B‐TCP, beta‐tricalcium phosphate; CT, computed tomography; GBR, guided bone regeneration; PCR, polymerase chain reaction; PICF, peri‐implant crevicular fluid; SFRP1, secreted frizzled related protein‐1; SLA, sandblasted, Large‐grit, acid‐etched; TNF‐a, tumor necrosis factor‐alpha.

## 5 FUNCTIONALIZATION OF DENTAL IMPLANT SURFACES WITH EPIGENETICS

**FIGURE 4:**
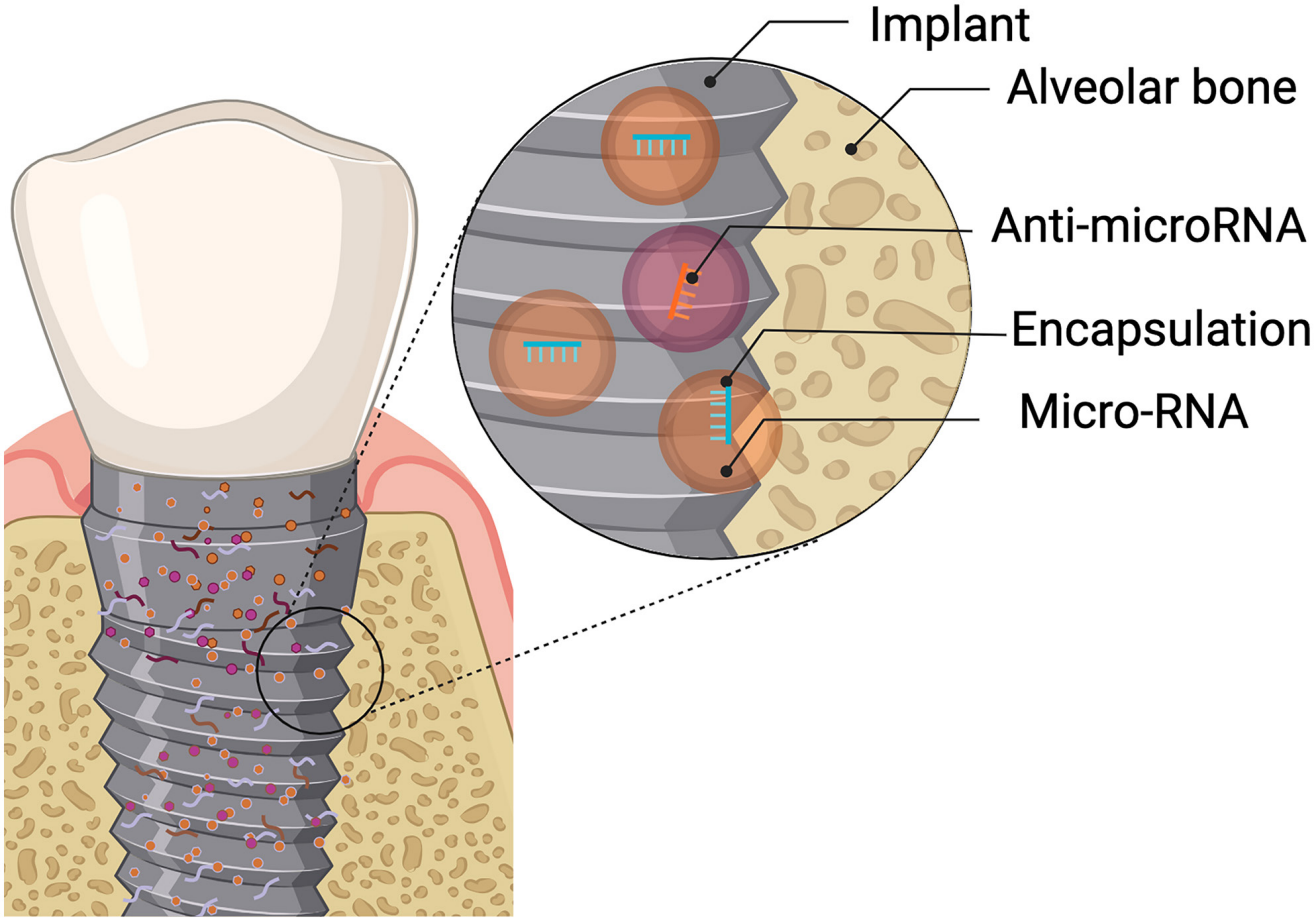
Functionalization of dental implant surfaces via epigenetic mechanisms.

Recent efforts have been devoted to identifying and developing innovative methods to improve osseointegration and reduce the incidence of implant failure. Predominantly, they revolve around altering the physiochemical characteristics of the surface of dental implants to promote osteoangiogenesis.^94^ As such, the application of epigenetic concepts within implant therapy holds promise in achieving these goals as discussed in a previous review.^14^ Nevertheless, scarce studies are available^95^, ^96^, ^97^, ^98^, ^99^, ^100^, ^101^, ^102^, ^103^, ^104^ and many of them have not investigated the use of histone modifications and DNA methylation, but instead, only the capability of miRNAs and antagomirs to coat implant surfaces (Figure 4).

According to Meng et al., a biodegradable coating was developed consisting of miR‐29b encapsulated in nanocapsules in an O‐carboxymethyl chitosan coating.^101^ To boost osteogenic bioactivity, the biodegradable coating underwent testing in a rat tibial defect model. The reason that the authors chose miR‐29b is due to its capacity to support osteoblast differentiation, inhibition of the function and differentiation of human osteoclasts, and directly reducing the expression of inhibitors of osteoblast differentiation, all of which stimulate osteogenesis.^105^, ^106^, ^107^, ^108^ Results from an in vivo model demonstrated that this coating exhibited superior characteristics in terms of cell adhesion and growth. Furthermore, it exhibited a substantial efficacy in miRNA transfection and osteoinduction resulting in a remarkable enhancement of bone regeneration around the titanium surface.^101^

MiR‐21 has also been investigated as a potential candidate for coating implant surfaces, owing to its extensively documented involvement in the regulation of cellular functions,^109^, ^110^, ^111^, ^112^ and its capacity to enhance the osteogenic differentiation of mesenchymal stem cells.^113^ In an in vitro study, a biodegradable composite coating on titanium implants that was made of strontium/hydroxyapatite loaded with miR‐21 nanocapsules facilitated osteoblast proliferation, differentiation, and mineralization.^97^ Moreover, in the same study, in vivo implantation experiments conducted on rabbits demonstrated that this coating stimulated the expression of the angiogenic factor CD31 and improved the expression of osteoblastic genes, thereby promoting osteoangiogenesis. The composite coating exhibited a reduced expression of receptor activator of nuclear factor kappa‐B ligand (RANKL) and consequently, facilitated the formation of new bone and mineralization leading to an enhanced osseointegration and improved strength in the bone‐implant bonding.^97^

Another example of the use of miR‐21 for implant surface coating was tested using biocompatible chitosan (CS) and hyaluronic acid (HA) nanoparticles developed specifically for the delivery of miR‐21 (also referred to as CS/HA/miR‐21 nanoparticles) into human BMSCs.^103^ The cross‐linking of the CS/HA/miR‐21 nanoparticles onto titanium surfaces treated with microarc oxidation (MAO) was conducted to create a miR‐21 functionalized MAO titanium surface. The in vitro findings demonstrated the positive impact of the implant coating on osteoblastic differentiation in human BMSCs. This was determined by the upregulation of early osteogenesis‐related gene markers, including Type I collagen (COL1), Type III collagen (COL3), RUNX‐2, osteoprotegerin (OPN), and osteocalcin (OCN). These results suggest that the coating holds clinical promise in enhancing osseointegration.^103^

MAO‐treated implants appear to be well‐suited for surface modification with miRNAs. In a study by Shao et al., a gene‐modified tissue‐engineered implant was developed by incorporating miR‐122‐modified cell sheets into MAO titanium implants.^100^ Their in vitro experiments demonstrated that miR‐122 facilitated osteogenic differentiation of bone marrow mesenchymal stem cell sheets.

The use of anti‐miRNAs, also known as antagomirs, has been suggested to alter the titanium surface. In a recent investigation conducted by Song et al., calcium ions (Ca^2+^) were used in an in vitro model to deliver small‐interfering RNAs (siRNA) and miRNA for the stimulation of osteogenic differentiation in human mesenchymal stem cells (MSCs) and enhance osseointegration.^98^ Given that miR‐138 has previously demonstrated its ability to inhibit focal adhesion kinase signaling, which plays a vital role in osteoblast differentiation, employing anti‐miR‐138 to suppress miR‐138 could potentially enhance the osteogenic differentiation of MSCs.^114^, ^115^ As such, calcium ions have been used to deliver anti‐miR‐138 into human MSCs from a nanotubular titanium implant surface.^98^ The results of this investigation demonstrated that implants functionalized with Ca^2+^/anti‐miR‐138 exhibited a remarkable enhancement in mineralization compared with the control groups and observed directly on the implant surface and surrounding areas. These findings hold potential significance for in vivo bone regeneration as they indicate enhanced osteogenesis of human MSCs in the vicinity of the functionalized titanium surface and adjacent regions.

Yan and co‐workers conducted additional evaluations of anti‐miR‐138‐functionalized titanium surfaces, both in vitro and in vivo.^99^ Findings from their in vivo study demonstrated the formation of robust vascularized bone indicating a promising coupling of osteogenesis and angiogenesis within the MSC sheet‐implant complex. This holds the potential for achieving osseointegration, particularly in situations where the bone condition is compromised. Moreover, MAO‐treated titanium implants have been evaluated for their osteogenic activity enhancement and inhibition of endogenous osteogenesis by functionalizing them with both miR‐29b and anti‐miR‐138.^104^ The results of this study revealed that the functionalized surface effectively stimulated the osteogenic differentiation of MSCs, as discovered by the upregulation of osteogenic markers, increased ALP production, collagen secretion, and mineralization of the extracellular matrix. Consequently, these miRNA‐functionalized titanium implants hold the potential to facilitate rapid and robust osseointegration at the bone‐implant interface.

Ultimately, Liu et al. investigated the modification of titanium surfaces with antagomirs, where they conjugated antago‐miR‐204 with gold nanoparticles (AuNP‐antago‐miR‐204).^102^ These conjugates were dispersed in a solution of PLGA and subsequently used to coat the surface of titanium implants by applying an ultrathin sheet aiming to promote osseointegration in rats with streptozotocin‐induced type 2 diabetes mellitus. The choice of targeting antago‐miR‐204 specifically was motivated by the initial identification of high expression levels of miR‐204 in the BMSCs of diabetic rats. The authors reported a successful release of AuNP‐antago‐miR‐204 from the PLGA sheet in the in vitro setup absorbed by the adherent BMSCs. The results obtained from the in vivo model demonstrated that this coating approach effectively enhanced osseointegration in rats with type 2 diabetes. Overall, these findings indicate that the PLGA sheet/AuNP‐antago‐miR‐204 coating strategy holds promise for improving osseointegration on titanium implant surfaces in patients with type 2 diabetes mellitus.

In a recent investigation by Zhang and colleagues (2023), a novel approach was undertaken involving the development of a titanium plate integrated with a laser‐drilled, metal organic framework‐miR‐27a agomir nanomembrane (L‐MOF‐agomir) implant.^95^ This innovative implant was engineered to facilitate the loading and controlled release of miR‐27a agomir. Through in vitro experiments, it was observed that the L‐MOF‐agomir implant effectively prompted the repolarization of macrophages stimulated by lipopolysaccharides (LPS), transitioning them from an M1 to M2 phenotype. Furthermore, the culture supernatant of these repolarized macrophages notably stimulated osteogenesis in BMSCs.

In additional experiments using a ligation‐induced rat peri‐implantitis model,^95^ the L‐MOF‐agomir implants demonstrated robust immunomodulatory effects on macrophage polarization and mitigated ligation‐induced bone resorption. The underlying mechanism of this repolarization effect is proposed to involve the promotion of macrophage mitochondrial function and metabolic reprogramming from glycolysis to oxidative phosphorylation by the L‐MOF‐agomir implants.

Findings of this study highlights the potential of targeting cellular metabolism to modulate macrophage‐mediated immunity for the inhibition of peri‐implantitis. Moreover, it offers a fresh perspective on the development of multifunctional implants with enhanced therapeutic capabilities.

A complete summary of studies evaluating functionalized implant surfaces can be found in Table 3.

**TABLE 3:** Characteristics of studies evaluating functionalized implant surfaces and their epigenetic changes.

Author	Type of study	Description/condition of sites	Type of implant surface	Epigenetic mechanism	Method	Findings
Wu et al.^104^	In vitro	MSC seeded onto a miRNA‐functionalized titanium disks	Titanium with microarc oxidation	MicroRNA expression	Real‐time PCR	Clear stimulation of MSC osteogenic differentiation with no apparent cytotoxicity was noted when functionalizing titanium surfaces with miR‐29b and anti‐miR‐138. MiRNA functionalized implants can lead to more rapid and robust osseointegration.
Wang et al.^103^	In vitro	BMSC cultured on microarc‐oxidized titanium surfaces	Titanium with microarc oxidation	MicroRNA expression	Real‐time PCR	Osteogenic differentiation of human bone marrow MSC was promoted when exposed to microarc‐oxidized titanium surfaces functionalized with miR‐21‐loaded chitosan/hyaluronic acid nanoparticles.
Meng et al.^101^	In vitro, In vivo	In vitro: Human umbilical cord MSCs cultured on functionalized titanium rods. In vivo: Effect of functionalized titanium rod upon tibial bone defect among Sprague–Dawley rats.	Titanium rods with CMCS/n(miR‐29b) coating	MicroRNA expression	Quantitative real‐time PCR	The functionalized coating system enhanced the osteogenic differentiation of human umbilical cord MSCs and promote bone regeneration of titanium alloy in vivo.
Liu et al.^102^	In vitro	In vitro/Experimental: BMSCs of diabetic rats exposed to a functionalized titanium surface	Titanium with AuNP‐antagomiR‐204 coating	MicroRNA expression	Quantitative real‐time PCR	In vitro: Encapsulated AuNP‐antagomiR‐204 were released from a PLGA sheet and uptaken by adherent BMSCs. Experimental: AuNP‐antagomiR‐204 released from PLGA sheet promoted osseointegration.
Geng et al.^97^	In vitro, In vivo	In vitro: Osteoblast‐like MG63 cells cultured on functionalized implant disks. In vivo: Functionalized titanium rods were implanted in New Zealand white rabbits.	Titanium‐based strontium‐substituted hydroxyapatite/miR‐21 composite coating	N/A	In vitro: Real‐time PCR, In vivo: Immunohistochemistry	In vitro: The functionalized composite coating (Titanium‐based SrHA/miR‐21) was beneficial for osteoblast proliferation, differentiation, and mineralization. In vivo: Functionalized coating promoted expression of CD31 and osteoblastic genes to facilitate angio‐osteogenesis.
Shao et al.^100^	In vitro	Rat BMSC cultured on microarc‐oxidized titanium surfaces	Titanium with microarc oxidation and miR‐122 cells sheets	N/A	Quantitative real‐time PCR	Functionalized implants with miR‐122 significantly enhanced, promoted, and accelerated osteogenic activity of bone marrow MSCs.
Song et al.^98^	In vitro	Human MSC exposed onto a functionalized implant surface	Titanium with calcium/anti‐miR‐138 nanotubular layer	N/A	Fluorescence microscopy	The functionalized implant strongly enhanced the osteogenic differentiation of human MSCs.
Yan et al.^99^	In vitro, In vivo	In vitro: MSC cultured onto a functionalized implant. In vivo: Subcutaneous implantation of MSCs sheet‐implant complex in 6‐week old immunocompromised mice.	Titanium with microarc oxidation and anti‐miR‐138 cell sheet	N/A	In vitro: Quantitative real‐time PCR, In vivo: Micro‐CT	In vitro: The functionalized anti‐miR‐138 implant surface greatly improved osteogenesis and angiogenesis. In vivo: Ectopic implantation assay revealed a robust vascularized bone formation, and it is anticipated to lead to rigid osseointegration.
Geng et al.^96^	In vitro	In vitro: MSC cultured in a functionalized implant surface. In vivo: Titanium cylinders implanted in femur and tibia among 60 New Zealand White rabbits.	Titanium with miR‐21 nanocapsules coating	N/A	In vivo: Micro‐CT, immunohistochemistry	In vitro: MiR‐21 promote angiogenesis, osteogenic differentiation, and enhanced osteoclastic activity of MSC. In vivo: miR‐21 nanocapsules coating accelerated vascularization, bone remodeling, and bone maturation resulting in a significantly improved bone‐implant contact/bonding strength.
Zhang et al.^95^	In vitro, Experimental	In vitro: Exposure of modified titanium plates coated with MOF‐agomir nanoparticles to BMSCs and macrophages. Experimental: Ligature‐induced peri‐implantitis among 3 groups (L, L‐MOF, L‐MOF‐agomir) among 60 Sprague–Dawley rats.	Laser‐drilled with a metal organic framework miR‐27a agomir nanomembrane	MicroRNA expression, Macrophage polarization	In vitro: Immunofluorescence, CCK8 assay Experimental: Micro‐CT	In vitro: L‐MOF‐agomir titanium plate promoted repolarization of LDP‐stimulated macrophages from M1 to M2 and the macrophage culture supernatant stimulated BMSCs osteogenesis. Experimental: L‐MOF‐agomir implants featured a strong immunomodulatory activity of macrophage polarization and alleviated ligation‐induced bone resorption.

Abbreviations: AuNP, gold nanoparticles; BMSC, bone marrow stem cells; CMCS, O‐carboxymethyl chitosan; CT, computed tomography; L, laser‐drilled; L‐MOF, laser‐drilled with metal organic framework; miRNA, microRNA; MSC, mesenchymal stem cells; PCR, polymerase chain reaction; PLGA, polylactic‐co‐glycolic acid; SrHA, strontium‐substituted hydroxyapatite.

## 6 FUTURE PERSPECTIVES FOR EPIGENETIC CHANGES ON CLINICAL DENTAL TREATMENTS

Despite a low incidence of early implant failure from most modern dental implants, an enhancement in osseointegration might represent a significant advantage for patients with systemic/behavioral conditions impairing wound healing (e.g., diabetes mellitus, smoking, and osteoporosis). Considering all the previous results together, it is possible to suggest that the incorporation of miRNAs or anti‐miRNAs into dental implant surfaces could play a significant role in enhancing osseointegration. Nonetheless, research on how surface topography and surface energy can affect the epigenome remains scarce.^66^

Clinical studies investigating the impact of novel surface modifications on epigenetics are insufficient and open a promising pathway for future studies to understand how deeply biomaterial surface modifications may influence the implant healing. Additionally, it remains unknown how the functionalization of dental implants with epigenetic factors can alter the behavior of the onset and pathogenesis of peri‐implant diseases. The treatment of peri‐implantitis represents a real challenge even for experienced clinicians and thus, the development of methods to prevent it would be of great significance. At present, the use of epi‐drugs is considered an attractive target for new treatment modalities of peri‐implantitis lesions. The importance of a complete understanding of these epigenetic mechanisms and the cellular functions behind them is necessary to maximize the outcomes of osseointegration and re‐osseointegration.

## 7 CONCLUSIONS

Current and experimental surface modifications for dental implants have demonstrated to elicit epigenetic responses in peri‐implant cells and influence osseointegration at a fundamental and preclinical level.

Emerging approaches in surface modifications for dental implants functionalized with epigenetic concepts have great potential with a significant impact on modulating bone healing during osseointegration stages. Future studies are necessary to assess how the functionalization of dental implants with epigenetics can impact the prevention, onset, pathogenesis, and disease progression of peri‐implant diseases.

## AUTHOR CONTRIBUTIONS

Marcel F. Kunrath conceived the main idea for the present manuscript. Marcel F. Kunrath and Farah Asa'ad contributed to the conception, design, writing, and critical review of the manuscript. Carlos Garaicoa‐Pazmino contributed to the design, writing, and critical review of the manuscript. Lena Larsson contributed to the writing, and critical review of the manuscript. Paula Milena Giraldo‐Osorno contributed to the writing of the manuscript, and the creation of figures. Aya Haj Mustafa contributed to the writing of the manuscript. Christer Dahlin contributed to the critical review and editing of the manuscript. All authors have read and agreed to the final version of the manuscript.

## FUNDING INFORMATION

The present work was supported by Hjalmar Svensson Foundation (HJSV2022048) to F.A. The authors do not have any financial interests, either directly or indirectly, in the products or information identified in the paper.

## CONFLICT OF INTEREST STATEMENT

The authors report no conflict of interest related to this review. The funders had no role in study design, data collection, data analysis, decision to publish, or paper preparation.

## Data Availability

Data sharing does not apply to this article as no new data were created or analyzed in this study.
